# Integrating pathway knowledge with deep neural networks to reduce the dimensionality in single-cell RNA-seq data

**DOI:** 10.1186/s13040-021-00285-4

**Published:** 2022-01-03

**Authors:** Pelin Gundogdu, Carlos Loucera, Inmaculada Alamo-Alvarez, Joaquin Dopazo, Isabel Nepomuceno

**Affiliations:** 1 Clinical Bioinformatics Area. Fundación Progreso y Salud (FPS). CDCA, Hospital Virgen del Rocio, 41013 Sevilla, Spain; 2 Computational Systems Medicine, Institute of Biomedicine of Seville (IBIS), Hospital Virgen del Rocio, 41013 Sevilla, Spain; 3 Bioinformatics in Rare Diseases (BiER), Centro de Investigación Biomédica en Red de Enfermedades Raras (CIBERER), FPS, Hospital Virgen del Rocío, 41013 Sevilla, Spain; 4 FPS/ELIXIR-es, Hospital Virgen del Rocío, 42013 Sevilla, Spain; 5 Department of Computer Languages and Systems, Universidad de Sevilla, Sevilla, Spain

**Keywords:** Deep neural network, Signaling pathway, Single cell, scRNA-seq, Gene expression, Transcriptomics, Machine learning

## Abstract

**Background:**

Single-cell RNA sequencing (scRNA-seq) data provide valuable insights into cellular heterogeneity which is significantly improving the current knowledge on biology and human disease. One of the main applications of scRNA-seq data analysis is the identification of new cell types and cell states. Deep neural networks (DNNs) are among the best methods to address this problem. However, this performance comes with the trade-off for a lack of interpretability in the results. In this work we propose an intelligible pathway-driven neural network to correctly solve cell-type related problems at single-cell resolution while providing a biologically meaningful representation of the data.

**Results:**

In this study, we explored the deep neural networks constrained by several types of prior biological information, e.g. signaling pathway information, as a way to reduce the dimensionality of the scRNA-seq data. We have tested the proposed biologically-based architectures on thousands of cells of human and mouse origin across a collection of public datasets in order to check the performance of the model. Specifically, we tested the architecture across different validation scenarios that try to mimic how unknown cell types are clustered by the DNN and how it correctly annotates cell types by querying a database in a retrieval problem. Moreover, our approach demonstrated to be comparable to other less interpretable DNN approaches constrained by using protein-protein interactions gene regulation data. Finally, we show how the latent structure learned by the network could be used to visualize and to interpret the composition of human single cell datasets.

**Conclusions:**

Here we demonstrate how the integration of pathways, which convey fundamental information on functional relationships between genes, with DNNs, that provide an excellent classification framework, results in an excellent alternative to learn a biologically meaningful representation of scRNA-seq data. In addition, the introduction of prior biological knowledge in the DNN reduces the size of the network architecture. Comparative results demonstrate a superior performance of this approach with respect to other similar approaches. As an additional advantage, the use of pathways within the DNN structure enables easy interpretability of the results by connecting features to cell functionalities by means of the pathway nodes, as demonstrated with an example with human melanoma tumor cells.

**Supplementary Information:**

The online version contains supplementary material available at 10.1186/s13040-021-00285-4.

## Background

High-throughput sequencing technology has revolutionized research in the area of biology and biomedicine. RNA sequencing (RNA-seq) allows the analysis of the entire transcriptome. However, RNA-seq data represents an average of gene expression values across thousands to millions of cells, i.e., is typically performed in “bulk” [1]. RNA-seq produces accurate count data allowing the detection of transcripts even at low expression levels [2] and also permits the detection of splicing and previously unknown transcripts [3]. RNA-seq has extensively and successfully been used to build prognostic gene signatures [4, 5] and other biomedical problems like location of regulatory elements [6]., the identification of disease-associated single nucleotide polymorphisms [7], and gene fusions [8]. Recent advances in RNA sequencing technologies have enabled the direct sequencing of individual cells, known as single cell RNA sequencing (scRNA-seq), which allows querying biological systems at an unprecedented resolution [9].

ScRNA-seq data provides valuable insights into cellular heterogeneity which may significantly improve the understanding of biology and human disease [10, 11]. One of the main applications of scRNA-seq data analysis consists of identifying new cell types and cell states [12, 13]. This application raises key questions to address, such as how to determine the similarity from expression profiles of cells or which cell types have an important role in diseased individuals. Consequently, two major computational challenges in this scenario are how to group cells and how to identify new cell types, i. e., clustering analysis and cell retrieval.

Clustering analysis consists of finding the closest cell/gene group to a sample given a population of cells. Aside from the data dimensionality problem (thousands of genes and samples), single cell data is polluted with high levels of noise from heterogeneous sources (gene dropout events, experimental and measurements errors etc.) To mitigate such problems, dimensionality reduction is usually performed before clustering. On the one hand, several unsupervised methods have been proposed to mitigate the influence of noise by reducing the dimension. Three of the most popular methods for this purpose are Principal Component Analysis (PCA) [14, 15], uniform manifold approximation and projection (UMAP) [16] and t-distributed stochastic neighbor embedding (t-SNE) [17]. PCA performs a linear reduction of the dimension leading to gene-based explainable models that lack the ability to capture the complex patterns behind single cell data, which can lead to poor performance or misleading interpretations in certain situations [15]. Instead, t-SNE and UMAP extract a low-dimensional representation of the data by means of non-linear methods that retains the similarities of the high-dimensional data but lacks direct biological interpretability even in the relationships between the clusters, although recent advances [18, 19] have corrected the cluster-based miss-interpretations (while retaining the global structure). Together with the three of the most popular methods for clustering analysis mentioned before, other novel methods have recently emerged based on the success of unsupervised Deep Learning to build a reduced number of features which capture cell heterogeneity and diversity, such as scCAEs [20], which uses the embeddings learned through convolutional autoencoders to feed a K-means-based clustering algorithm, scDeepCluster [21], which learns a latent representation by explicitly modelling the scRNA-seq data generation process, and scvis [22], that uses a feed forward neural network to learn the parameters of a reduced statistical representation of the gene expression space. On the other hand, motivated by the unprecedented success of Deep Learning in supervised tasks, a method to perform dimensionality reduction based on neural networks (NN) has recently been proposed [23]. This method combines a NN model with a protein-protein interaction (PPI) network to classify several cell types. This model is trained in a supervised way and after that, the last hidden layer of the network is used as a low dimensional representation of scRNA-seq data, which can be used for cell retrieval analysis. Cell retrieval or cell type annotation consists of inferring the cell type of a given cell by querying a reference database of annotated scRNA-seq data. For this challenge, traditional supervised methods such as Support Vector Machine (SVM) or Random Forest classifier (RF) are often time-consuming [24] due to the huge size of the aforementioned reference databases.

In this work, we integrate prior biological information in network architectures as a way to reduce the dimensionality of the scRNA-seq data in a supervised framework in order to learn a reduced representation of the data that can be used for cell retrieval, unsupervised clustering and biological knowledge extraction. Actually, the introduction of biological information into the structure of machine learning models has recently been recognized as an important asset to improve prediction accuracy and increasing model interpretability [25]. In particular, we propose the use of signaling pathways, a specific type of biological networks related to the knowledge available on cell functionality. Pathways can be found in repositories such as KEGG [26], and are represented as graphs that encode the biological knowledge on the complex relationships among proteins that allow them to carry out the functions that permits the survival of the cell, its proliferation, differentiation into distinct cell types, interaction with the environment, and many other biological processes. Pathways have already been used in the context of machine learning based cancer classification [27, 28]. Unlike the limited functional information provided by other types of biological networks, like physical proximity, encoded in protein-protein interaction (PPI) networks, or direct gene activation, encoded in gene regulation networks (GRN) used in a previous approach [23], the relationships among proteins encoded in pathways link protein activity with cell phenotype and behavior, which makes them ideal for distinguishing between cell types. Thus, including in the DNN architecture pathway knowledge allows obtaining a smaller architecture (less nodes and hence faster inference), which is easier to interpret [25] and that performs as well as other methodologies in a set of cell type identification benchmarks.

Moreover, the analysis of the biological relevance of the learned representations in a set of cells belonging to different melanoma tumors found an excellent agreement with what is already published in the literature. Therefore, the proposed model constitutes a highly efficient strategy to precisely identify cell types in scRNA-seq data.

## Results and discussion

To evaluate the performance of the proposed model four complementary analyses were carried out: 1) several unknown cell-type scenarios were simulated to check the ability of the model to properly cluster the new cell types, 2) the DNN were trained using a small database of single cells and annotate a bigger collection of unseen cells (the so-called retrieval analysis), 3) the unsupervised visualization capabilities of the representation learned by the model was demonstrated, and finally 4) the biological interpretability of the pathway-primed network was assessed. For the first and second steps, the validation schemes previously discussed in Materials and Methods were followed, which allowed the comparison of this proposal with previous proposals [23] in the same terms, using the same data (a collection of mouse single cell datasets) and splits. For the third and fourth steps, the melanoma dataset was used following the data splits (labelled as training and testing) defined in the original publication [29], allowing to check performance of our approach. Table 1 describes the architectures used here. See Supplementary Table 1, Additional File 1 for a complete list of the datasets, their public accession codes and how to combine them. However, before the performance of our model was benchmarked in *unsupervised* tasks, a more classical *supervised* performance experiment was carried out.

**Table 1:** Details of input and hidden layers, as well as parameters for each architecture used

Dataset	Architecture	Nodes Layer 1	Nodes layer 2	Effective parameters (million)
	Dense	100	–	0.95 M
	Dense + pathway	100 + 92	–	0.95 M
	Dense + PPI	100 + 348	–	0.96 M
Mouse	Dense + PPI and GRN	100 + 696	–	1.01 M
	Dense + PPI and GRN	100 + 696	100	1.08 M
	Pathway	92	–	0.01 M
	Pathway	92	100	0.02 M
	Dense	100	–	1.80 M
Human	Pathway	93	–	0.01 M
	Pathway	93	100	0.02 M

### Architecture, parameters and hyperparameter selection

Several activation functions (tanh, relu and sigmoid) and prepossessing steps (normalization, log-transform and [− 1, 1]-scaling) for the DNN were tested. The normalization and tanh were the better options for the mouse experiment, which is congruent with previous findings [23]. Regarding the preprocessing steps, the combination of relu with logarithmic pre-processing worked better than other approaches in the human melanoma dataset. Detailed information about the number of parameters and the dimensions of each architecture is shown in Supplementary Table 2, Additional File 1 and Table 1, respectively.

### Clustering and supervised performance

To test the performance of the learned representation of the proposed DNN, it was compared against similar dimensionality-reduction-based clustering approaches. The analysis consists of the following multi-step simulation: 1) combine a set of different mouse single cell experiments into a single dataset (the so-called *learning* set), 2) keep only those genes that appear in all the included experiments, 3) conduct a supervised analysis and 4) simulate the clustering of unknown cells.

**Fig. 1:**
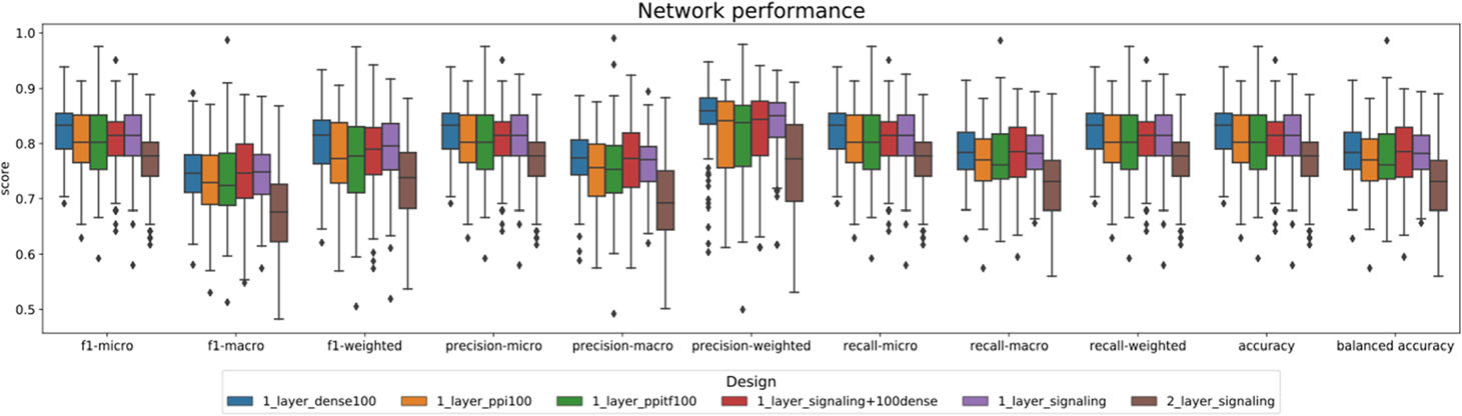
Network performance. The figure depicts the global metrics distribution for each design in a supervised task scenario. It shows the cell types prediction i.e., the performance of different models following a 100-times repeated stratified holdout cross-validation schema with a test size of 0.30

Table 2 provides a comparison of the performance of different models for supervised tasks following a 100-times repeated stratified holdout cross-validation validation schema with a test size of 0.30. Under this scheme, the model learns how to predict the cell types (the output) from the cell’s gene expression profiles. The class distribution can be consulted in Supplementary Table 3, Additional File 1: a clearly unbalanced set. For each realization, the accuracy, the imbalanced-aware balanced accuracy, precision, recall and F1 scores were computed. Although there were 16 classes to predict with several underrepresented cell-types, the results were very good (and equivalent) for all the models, with mean F1 scores above 0.8. The performance of the classification was similar to the supervised performance reported [23]. Figure 1 shows the global metrics distribution for each design, whereas Supplementary Fig. 1, Additional File 1 shows the per-class metric distributions, where it is clear that the lower sampled classes clearly cause a decline of the all-class scores.

**Table 2:** Average performance (F1, accuracy, precision, recall) of the different models in a supervised task scenario. Although our pathway-primed models are nearly ten times smaller (sparse), the performance is very close to the PPI-based NN. We report the mean for 100 iterations of train test splits

				F1			PRECISION			RECALL		
**Architecture**	**Number of nodes** **(2nd hidden layer)**	**Accuracy**	**Balanced accuracy**	**Macro**	**Micro**	**Weighted**	**Macro**	**Micro**	**Weighted**	**Macro**	**Micro**	**Weighted**
Dense	–	0.825	0.788	0.748	0.825	0.802	0.769	0.825	0.844	0.788	0.825	0.825
Dense with pathways	–	0.810	0.781	0.743	0.810	0.783	0.763	0.810	0.823	0.781	0.810	0.810
Dense with PPI	–	0.802	0.770	0.730	0.802	0.774	0.753	0.802	0.817	0.770	0.802	0.802
Dense with PPI/GRN	–	0.800	0.777	0.735	0.800	0.771	0.757	0.800	0.815	0.777	0.800	0.800
Signaling pathways	–	0.813	0.781	0.743	0.813	0.790	0.764	0.813	0.834	0.781	0.813	0.813
	100	0.766	0.724	0.673	0.766	0.728	0.690	0.766	0.762	0.724	0.766	0.766

However, the previous validation procedure does not properly address the key questions in scRNA-seq, such as how to identify unseen cell types or finding the most similar known cells to a new set of previously uncharacterized cells. To overcome the limited conclusions of a conventional supervised testing approach, the validation scheme described in Materials and Methods was followed, where each network is evaluated for randomly selected left out cells (LPGO) for P equal to 2, 4, 6, or 8 cell types of the 16 cell types (repeated 20 times for each P). Therefore, each selection of P leads to a clear division of train and test splits, where the knowledge-primed models can be safely trained under a supervised modality using the train split and then an independent encoding can be computed (see “Materials and methods” section) for the test set. Thus, by definition, none of the P cell types of the test have been seen by the model before, which ensures a fair comparison between the models in terms of the clustering performance using the K-Means algorithm (see “Materials and methods” section). For each realization, the following scores (higher is better) were computed: homogeneity, completeness, V-measure, adjusted random index (ARI), adjusted mutual information (AMI) and Fowlkes-Mallows. In addition, each realization was summarized by means of the average of all the previous scores.

**Fig. 2:**
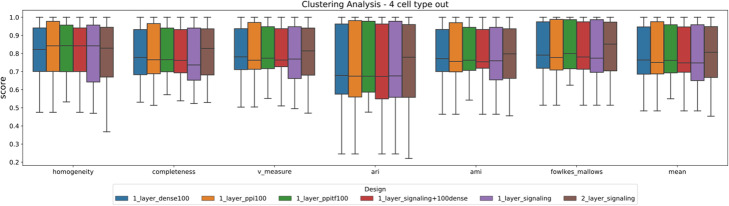
Clustering performance in the 4-left-out experiment. Each network is trained by leaving 4 cell types out (LPGO technique). The cell types which are left-out are randomly selected, and the procedure is repeated 20 times. After the neural network training is completed, the encoding (learned representation) is computed for the test (left-out cells) and used as input to the K-Means algorithm. The output is then evaluated using a comprehensive set of metrics (see “Materials and methods” section)

Table 3 provides a summary (the mean for each metric) of the results for the LPGO (*P* = 4) analysis. For a complete report (all P) refer to Supplementary Table 4, Additional file 1 and Fig. 2, which show the distribution of the different metrics across the different realizations. In all cases the pathway-based NN offers a comparable performance to the less interpretable PPI- and GRN-based models and the baseline dense NN.

**Table 3:** Unknown cell-type clustering performance of the different models analyzed for the LPGO experiments (*P* = 4). Although our pathway-based models are nearly ten times smaller (sparse), the performance is very close to the PPI-based NN. The mean of 20 splits was reported

Architecture	Number of nodes (2nd hidden layer)	Homogeneity	Completeness	V-measure	ARI	AMI	Fowlkes-Mallows	Average
Dense	–	0.801	0.799	0.798	0.725	0.786	0.814	0.787
Dense with pathways	–	0.804	0.797	0.798	0.718	0.786	0.811	0.786
Dense with PPI	–	0.811	0.804	0.805	0.728	0.794	0.817	0.793
Dense with PPI and GRN	–	0.820	0.808	0.812	0.746	0.802	0.827	0.802
Signaling pathways	–	0.797	0.788	0.790	0.716	0.778	0.809	0.780
Signaling pathways	100	0.775	0.803	0.786	0.729	0.774	0.820	0.781

### Retrieval analysis

Cell type assignment or annotation is one of the most important tasks in single cell expression analyses since each study usually profiles several different types of cells [30]. However, cell-type retrieval is also a major challenge due mainly to marker absence, overlap and noise-based problems when computing gene expression. Thus, the cells need to be clustered and compared with reference pre-annotated databases. Although groups of cells can be successfully grouped using unsupervised methods, it might not be easy to find patterns while clustering, leading to cell groups that are difficult to interpret from a biological point of view. In this work, an unsupervised biologically meaningful encoding was extracted of new data by leveraging the representation and comprehension capabilities of supervised neural networks with the interpretability of pathway-based gene clusters, which is in line with the recent literature where it is observed that supervised cell type assignment/annotation of newly-generated data using annotated labels has become more desirable than unsupervised approaches [25, 29].

In this work, two datasets that simulate the problems previously outlined (see “Materials and methods” section) were constructed: for the mouse dataset, the so-called *learning* set is used for training and optimizing the different learning methods (i.e. the pre-annotated database), whereas the *retrieval* set is comprised of new cells extracted from different experiments. For each cell in the latter set its encoded representation is computed using a model fitted with the former set and the most suitable cell type is retrieved by using the top *k = 100* matches. Table 4 shows the performance in terms of the Mean Average Precision (MAP) for each cell type and method. In contrast with the previous experiments, the knowledge-based networks need the addition of dense nodes in order to achieve a performance comparable to PCA. However, the biological priors are still relevant since the average performance of knowledge-based networks adding dense nodes is higher than the baseline dense network. Note that the validation scheme provides a fair comparison between supervised and unsupervised models since the cells of the retrieval set are encoded using pre-fitted models that could never use the ground truth cell types. Also, Supplementary Fig. 2, Additional File 1 shows a visualization of the encoding representation of the retrieval set.

**Table 4:** Average retrieval performance across the different cell type

Architecture	Number of nodes (2nd layer)	HSC	4cell	ICM	Spleen	8cell	Neuron	Zygote	2cell	ESC	Mean
PCA 100 (with full gene space)	–	0.181	0.669	0.026	0.975	0.176	0.627	0.462	0.675	0.106	0.433
PCA 100 (with signaling gene space)	–	0.179	0.561	0.128	0.989	0.191	0.624	0.455	0.676	0.205	0.445
Dense	–	0.243	0.643	0.000	0.734	0.147	0.404	0.569	0.514	0.148	0.378
Dense with signaling pathways	–	0.259	0.648	0.000	0.849	0.236	0.486	0.509	0.656	0.130	0.419
Dense with PPI	–	0.196	0.638	0.041	0.927	0.212	0.550	0.575	0.686	0.179	0.445
Dense with PPI/GRN	–	0.194	0.645	0.007	0.930	0.294	0.542	0.600	0.711	0.190	0.457
Dense with PPI/GRN	100	0.068	0.771	0.182	0.956	0.849	0.561	0.415	0.553	0.710	0.563
Signaling pathways (+)	–	0.163	0.438	0.011	0.619	0.179	0.475	0.344	0.465	0.128	0.314
Signaling pathways (+)	100	0.149	0.307	0.050	0.402	0.198	0.352	0.592	0.368	0.137	0.284
Signaling pathways (parameter tuning) (+)	–	0.107	0.768	0.049	0.960	0.625	0.549	0.471	0.627	0.110	0.474
Signaling pathways (parameter tuning) (+)	Size*	0.155	0.803	0.117	0.955	0.568	0.550	0.497	0.623	0.150	0.491
scvis	–	0.203	0.522	0.000	0.813	0.077	0.733	0.424	0.683	0.512	0.441

*The size of second layer (size) is defined after tuning in hyperparameter tuning networks

Furthermore, the optimized versions of the pathway-based models that do not contain dense nodes (see “Materials and methods” section) are on par with PCA, scvis [22] and the DNN designs that use dense nodes. A remarkable achievement since the non-dense architectures are sparser (~ 50 times) than the networks that depend on the addition of dense nodes for the first hidden layer. In addition, there are slight increases in the average performance when using deeper optimized models: from 0.474 to 0.491 in the pathway-based network (see Table 4). It is important to note that best scoring networks use a biological layer free of dense nodes, which makes them easier to interpret. Note that this kind of network design is marked with a “+” sign in the tables.

### Encoding visualization and functional analysis in melanoma dataset

One of the key advantages of the method presented is the ability to use the model for clustering analysis while retaining a sense of the underlying biological meaning of the learned weights. The biological interpretation can be inferred from the *pathway* activation scores of the first hidden layer, whereas the clustering is performed by computing the activation values of the last hidden layer, which can be used for data visualization by coupling it with a 2D reduction method (t-SNE in this case).

**Fig. 3:**
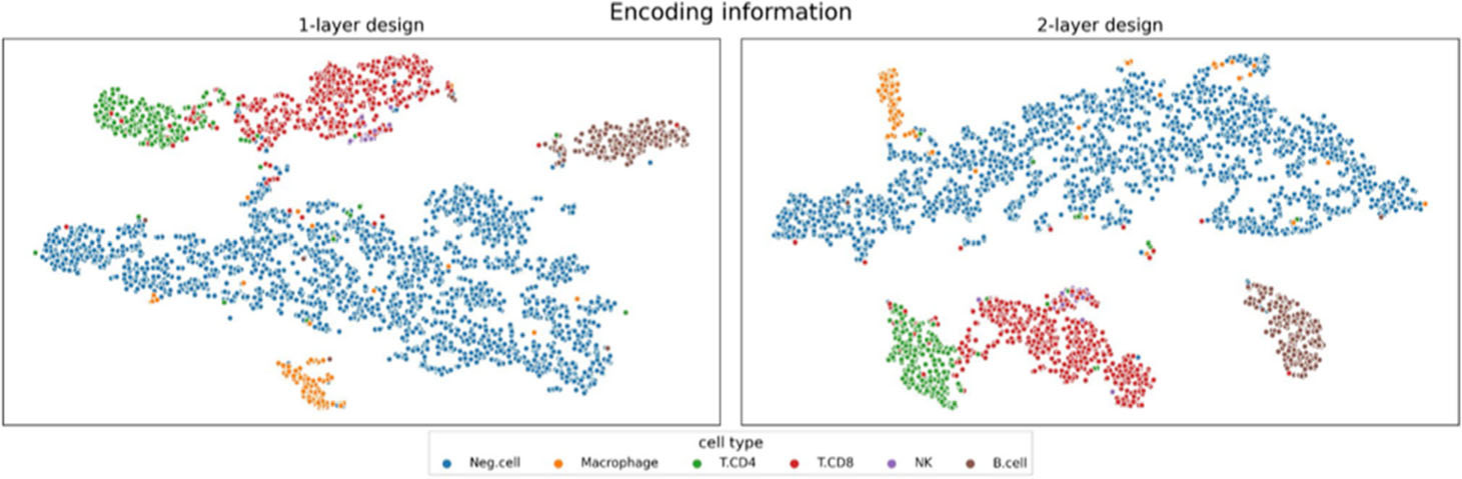
2D TSNE showing the dimensional reduction result based on the learned representation (encoding) of the data. Each pathway-based NN (1 and two layers designs) was trained using the training set and then used to compute the encoding of the training and test sets

To check the visualization and biological intelligibility capabilities of the proposed pathway-based DNN, a recent human melanoma dataset [31] comprising 33 human melanoma tumors from 31 patients, with more than 17,000 genes and 2761 single cell expressions, in which 5 different cell types were profiled, was analyzed. To fairly evaluate the proposed model, it was tested using the data splits (labelled as *training* and *testing*) defined in the original publication [31]. The model rendered an excellent performance (see Table 5 for the overall metrics and Supplementary Fig. 3 for the per-class metrics), with F1-scores above 0.9, comparable to previously reported metrics [23]. In addition, the clusters found by the model in the *testing* set can be visualized in Fig. 3 for either the one- or two-layer designs with pathways as prior biological knowledge. In all cases, the *training* set was used for fitting any given model, whereas the *testing* set has been used for checking the performance (supervised, visualization and interpretability). The cell-type distribution of both splits can be consulted in Supplementary Table 5, Additional File 1.

**Table 5:** Proposed network performance with log normalization

				F1			PRECISION			RECALL		
Architecture	Number of nodes 2nd layer	Accuracy	Balanced accuracy	Macro	Micro	Weighted	Macro	Micro	Weighted	Macro	Micro	Weighted
Dense	–	0.938	0.839	0.859	0.938	0.934	0.923	0.938	0.938	0.839	0.938	0.938
Pathways	–	0.936	0.844	0.861	0.936	0.933	0.922	0.936	0.938	0.844	0.936	0.936
Pathways	100	0.930	0.834	0.847	0.930	0.926	0.901	0.930	0.932	0.834	0.930	0.930

A functional analysis using the 10 highest-weighting groups (hidden nodes) for each cell type was performed. Since the nodes represent pathways, the functions of the pathways represented by those nodes were assessed. Supplementary Table 6, Additional File 1 summarizes the top pathways related to each cell type. The learned NN associates the 5 cell types with some very relevant pathways for the specific functions that are performed by these cell types.

For example, B cells mediate the production of antigen-specific immunoglobulin (Ig) against pathogens. The most important pathways for B cell functioning would be those engaged in cell-to-cell communication, proliferation, protein expression, and secretion. The pathways that appear among the 10 most relevant only for B-cells are: *Aldosterone-regulated sodium reabsorption* (hsa04960), *Pancreatic secretion* (hsa04972), *Complement and coagulation cascades* (hsa04610) and *Ovarian steroidogenesis* (hsa04913). *Complement and coagulation cascades* (hsa04610) consist of a nonspecific defense mechanism against pathogens. Although the complement system works in innate immunity, complement effectors are engaged with humoral immunity at multiple stages of B-cell differentiation and can influence B-cell biology on several levels through the complement receptors that they express [32]. The rest are pathways mainly involved in the secretion of substances and ion flow through the cellular membranes. It is expected in B cells that modules engaged in secretion processes are active, given that they expand their secretory organelles in their differentiation process [33].

Macrophages are innate immunity specialized cells that detect, phagocyte and destroy pathogens and debris. They can interact with the adaptive immune system by presenting antigens to T cells, and are secretory active, since they release cytokines and other substances that modulate the activities of other cell types. Among the most relevant pathways for macrophages alone, we can find *PI3K-Akt signaling pathway* (hsa04151) and *Osteoclast differentiation* (hsa04380). In the first one converge inflammatory and metabolic signals that are very relevant for the regulation of macrophage responses modulating their activation phenotype [34, 35]. The second one is relevant because both osteoclasts and macrophages derive from the monocyte-macrophage lineage, and monocytes use receptor/ligand systems that share signaling mechanisms with those used by immune cells [36].

Furthermore, the GO analysis of the active genes in the top 10 pathways in NK cell types reported the following enriched GO Terms related to immunity response: GO:0002228 (natural killer cell-mediated immunity), GO:0002443 (leukocyte-mediated immunity) and GO:0002449 (lymphocyte-mediated immunity).

The GO analysis of the active genes in the top 10 pathways in TCD8 and TCD4 cell types reported also enriched GO Terms related to T-cell proliferation and cytokine production: GO:0042098 (T cell proliferation), GO:0050852 T cell receptor signalling pathway) and GO:0042110 (T cell activation).

There are also several pathways that appear among the 10 highest-weighting nodes for 4/5 of the cell types. In particular, it is worth mentioning *Serotonergic synapse* (hsa04726). Although we are not working with nervous system cells, immunological and nervous systems share molecular and functional properties, such as cytokine networks and cell surface receptors [37, 38]. Synapses are interfaces between cells that transfer information from one cell to another, and in the immune system we can find somewhat similar processes: immunological synapses and kinapses [39].

Another pathway that is relevant throughout cell types is *Taste transduction* (hsa04742). The role of taste receptors in the immune response has been widely discussed and their expression has been reported in peripheral lymphocytes [40], NK cells [41], and macrophages [42].

The aim was to shed some light on how the gene expression of the genes contained in these pathways contribute to the algorithm being able to discern these cell types, but, overall, among the top relevant pathways, many are related to the molecular mechanisms expected most active in immune cells, or contain mechanisms common to the ones expected in their physiological functioning.

## Conclusions

This work demonstrated how the integration of pathways with neural networks can be used to learn a biologically meaningful representation of scRNA-seq data. Comparative results provided evidence of the good performance of integrating pathways as prior knowledge with neural networks, using smaller architectures than other NN-based approaches. The proposed approach obtained comparable results while being a more interpretable model thanks to the use of signaling pathways as the biological priors of the model since they provide a curated set of specific functions.

Furthermore, the biological relevance of the model learned on the melanoma research problem was also evaluated using the human scRNA-seq data [43]. Functional analysis of the results revealed associations between the 5 cell types and several relevant pathways for the specific functions that are performed by these cell types.

## Materials and methods

### Datasets

The DNN architecture was evaluated in two different scenarios: 1) a combination of different *Mus musculus* single cell experiments used for a comparison with other clustering and cell type annotation methodologies by means of the benchmarks [24], and 2) a collection of single cell experiments of different human melanoma tumors [29] used for the biological validation of our model.

### Mouse dataset

Single cell gene expression data from several mouse tissue sites are downloaded from the NCBI Gene Expression Omnibus (GEO) [44] database. There are more than 17,000 single cell expression profiles gathered in 33 datasets from different experiments and laboratories (see Supplementary Table 1, Additional File 1 GEO_IDs).

Data has been grouped into two sets of samples, namely *learning* and *retrieval* dataset. The *learning* dataset integrates three *Mus musculus* scRNA-seq datasets, which contains 9437 genes and 402 cells involving 16 cell types. This dataset is used for training the supervised models and for clustering analysis. The *retrieval* dataset was created by joining 31 datasets with more than 17,000 single cell expression profiles. It is used during the retrieval analysis to test the proposed pathway-based DNN (see below) over an independent set. Moreover, this dataset includes cell types that do not exist in the *learning* dataset.

### Human dataset

This dataset integrates multiple datasets (see [31]) from public repositories such as Broad Single Cell Portal, Gene Expression Atlas - EMBL-EBI [45], NCBI Gene Expression Omnibus [44] and CellBlast [46]. We use the exact *training* and *testing* divisions, as previously proposed [29], which can be downloaded from their companion website [43]. Note that the dataset is already normalized using Transcript Per Million (TPM) [47].

The *training* dataset has more than 17,000 genes and 2761 single cell expressions for 5 different cell types (B-cell, Macrophage, NK, T.CD4, T.CD8), after removing the malignant, cancer-associated fibroblasts and endothelial cells. The *testing* dataset is used to evaluate the performance of the supervised network and clustering analysis. This dataset has 3415 samples, single cell expression profiles from 6 different cell types (B-cell, Macrophage, NK, T.CD4, T.CD8 and Neg. cells). These two datasets are imbalanced datasets and NK is the least representative cell type for both datasets. Note that the *testing* dataset has one additional unseen cell type. We applied log-normalization (*log*(1 + *x*)) to both datasets.

### Sources of biological information

Although the predictive power of Deep Learning methods is enough to justify its use, the explainability of machine learning models is a desirable goal [25]. By incorporating prior biological knowledge, a double aim is pursued: i) to have an architecture in the NN that captures the way in which proteins interact among them to define the phenotypes we seek to characterize and ii) to provide a way to interpret the underlying biological mechanism behind the DNN-based method. In this work, signaling pathways, as described in KEGG [26], were used as the main source of biological information to constrain the network architecture. Pathways were used to define function-driven curated clusters (i.e. clusters of genes grouped by a biological common functionality using pathways). In order to compare the use of signaling pathways with other types of biological information previously used [23] the genes have also been grouped by PPIs and GRNs (see Table 6).

**Table 6:** Number of nodes in the input layer (genes) and in the first hidden layer (biological information) according to the type of biological information used to relate genes among them

Organism	Biological information	Source	Number of genes (input layer)	Number of nodes (first hidden layer)
	PPI	[23]	3553	348
Mouse	GRN	[29]	8307	348
	Signaling pathway	[26]	3737	92
Human	Signaling pathway	[26]	2987	93

### Neural network design

The neural network proposed here consists of one *input* layer, one or two *hidden* (intermediate) layers and one *output* layer connected between them by a set of weights. The *input* layer ciphers the gene expression values, whereas the *output* layer encodes the probability of each cell type, which is learned as the information is propagated throughout the intermediate layers back and forward, updating the weights at each iteration (the so-called *epochs*). In the end, the network learns an internal representation of the underlying function of the data which in our case is conditioned by the biological priors used to construct the first hidden layer.

The neural network model is formulated as follows:

$$ {x}^i=a\ \left(\ {W}^i\ast {x}^{\left(i-1\right)}+{b}^{\left(i-1\right)}\right) $$

where *x*^*i*^ denotes the activation score in *i* th hidden layer, *a* is the activation formula, *b* is bias value and *W* is the weight matrix (the *edge*s of the neural network). The activation function for each hidden layer is either *tanh* for all the mouse single cell experiments, or *relu* activation for human data. Finally, the *softmax* activation function is used in the output layer.

$$ \mathit{\tanh}(x)=\frac{1-{e}^{\left(-2x\right)}}{1+{e}^{\left(-2x\right)}} $$

$$ relu(x)=\mathit{\max}\left(0,x\right) $$

$$ softmax\left({x}_i\right)=\frac{e^{x_i}}{\sum_{j=1}^n{e}^{x_i}} $$

Since the aim was to solve a (classification) *supervised problem*, where the outputs to learn (the classes/labels) are the different cell types, the *cross-entropy loss* was minimized as it is typically done in the literature. The *cross-entropy loss* is defined as:

$$ -{\sum}_{c=1}^M{y}_{o,c}\mathit{\log}\left({p}_{o,c}\right) $$

where *M* refers to the total number of cell types, *y* is a binary indicator if cell type *c* is the correct label for a sample *o* and *p* is the predicted probability of observation *o* for cell type *c*. Note that *o* traverses the sample space.

### Prior biological information integration

In order to incorporate the biological priors, the first hidden layer was adjusted in two ways: 1) each neuron/node corresponds to one biological unit, in this case there are as many neurons as pathways and 2) the weights that arrive to a neuron are fixed to zero when no input gene participates in the pathway associated to the node. In this way, biological priors were incorporated using known gene clusters with defined functions (the pathways) at the same time that the size of the model is reduced, which can help with over-fitting as well as training and inference time.

### Architectures, parameters and hyperparameter selection

A total of 6 architectures were tested: with and without biological knowledge, and either 1-layer or 2-layer options. See Table 1 for a detailed summary of the different architectures. All models were implemented using the Keras API of Tensorflow [48], with the pertinent modifications over the dense layer definition to include the *biological* nodes. The models were trained using stochastic gradient descent (SGD) for mouse analysis and the Adam optimizer for human analysis, using Glorot initialization [49] and 100 training epochs with a mini-batch size of 10 (see Supplementary Table 2, Additional File 1). Several activation functions (tanh, relu, linear and sigmoid) and prepossessing steps (normalization, log-transform and [− 1, 1]-scaling) were also tested.

Additionally, we tested how the hyperparameter chosen can help to increase the performance of the model by using the hyperband method [50] to optimize the learning rate, momentum and decay of the SGD. The test was conducted using the mouse retrieval benchmark with remarkable gains in performance. The hyperband method provides an improvement over random search by reaching a trade-off between whole-space (global) exploration and local exploitation thanks to a guided hyperparameter sampling which allocates most resources into promising regions, by iteratively exploiting *smaller* neighborhoods of promising configurations, while still performing random sampling in parallel. A parallel worker constantly evaluates the proposed configurations in order to check for early stopping conditions. Note that the independence of the hyperparameter optimization and performance evaluation is ensured since the cell type retrieval task is composed of two independent sets of datasets: the *learning* dataset is used to optimize and train the model while the *retrieval* set is used for checking the performance. This validation schema also allows fair comparisons between the optimized and non-optimized models over the same set of cells.

### Encoded information

**Fig. 4:**
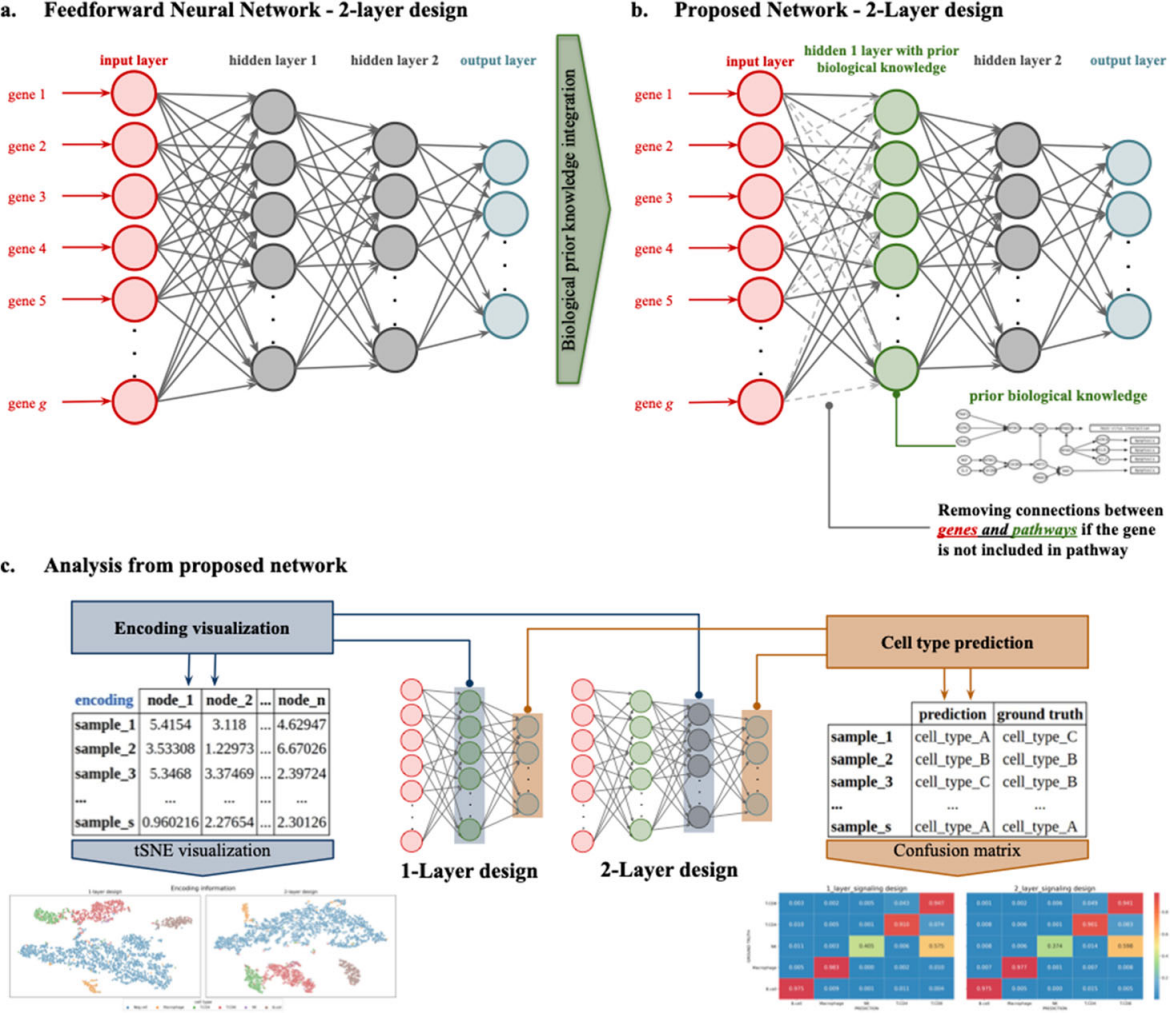
Encoding information in the DNN. a. The proposed network is based on a feedforward neural network. b. The integration of biological knowledge is implemented into the first hidden layer, i.e. each neuron/node corresponds to one biological unit. c. The learned representation or encoding (the activation of the last hidden layer) can be used as the input of the TSNE algorithm to produce a 2D visualization of the data. Finally, the supervised performance of the network can be evaluated with classical ML metrics

As mentioned before, while the NN learns how to predict any given cell type seen during the training, the intermediate layers learn a representation of the problem, which can be used for unsupervised tasks by detaching the output layer during the inference. Thus, with only one trained model both, unsupervised and supervised tasks are solved, by either computing the activation values of the last hidden layer or using the whole model (predicting the activation of the output nodes), respectively. Furthermore, if the computation is stopped at the first hidden layer the activation values of the *biological* nodes are obtained, which can be translated into pathway activities, which can be summarized by any cell group, e.g. cell types. Finally, the weights that connect the *biological* layer with the input can be further inspected to understand the role played by each gene when inferring the pathway activities of any given group of cells. Figure 4 summarizes the encoding of the network.

### Validation methodology

To compare the proposed approach with the relevant literature, the validation schemes already proposed [23], which consist of (1) simulating how a method clusters unseen cells and (2) cell type retrieval from a large database was implemented.

In order to check the performance of the proposed method when clustering unseen cells (with respect of where the model is trained) a 20 times repeated leave *P* groups out (LPGO) cross-validation scheme for *P ∈ (2,4,6,8)*, where the groups are defined by the cell types, was performed in the mouse dataset. During each run, the method is tested in a fold which contains all the cells that belong to *P* cell types and fitted with a training fold composed of samples of the complementary groups. Once a model is trained, the output layer is detached and the learned representation of the testing samples is computed. That is, the single cells were encoded into a reduced space, which are finally clustered using K-Means. Then, different metrics are used to compare the true labels (never seen by the model) with the clusters identified by the proposed method.

In addition to the unseen cell type clustering simulation, a retrieval analysis experiment was carried out, which involves inferring the cell type of a sample by querying a reference database of annotated scRNA-seq data. For this purpose, the model was fitted using the full *learning* set. Then, the unsupervised capabilities of the model were used to obtain the encoded representation of the *learning* and *retrieval* sets of cells. To measure the performance of cell retrieval, the *k* = 100 nearest neighbors (using the euclidean distance between the encoding representations) of the learning set to each *retrieval* sample were computed. Finally, the performance was checked by computing the MAP of the matches.

Both validation schemes closely match those presented in a previous work [23], making the results of this approach completely comparable to the different methodologies already benchmarked there. In fact, as a safeguard, the PCA-based methods were re-implemented and the results obtained were the same. In addition, we have also benchmarked the scvis method (using the default hyperparameters), a state of the art deep-generative model for single cell dimensionality reduction [22].

### Encoding visualization

As a side effect of the unsupervised capabilities of the proposed model, the learned representation of the data can be used to visually inspect the structure of the data by making use of two-dimensional unsupervised transformations of the learned features (e.g TSNE, PCA, etc.) In particular, the learned representation is the encoded information obtained after computing the activation values of the last hidden layer and detaching the output layer from the model. The learned feature dataset constructed from the encoded information (see Fig. 4) can be used as input to the TSNE or PCA algorithm mentioned above to visually inspect the structure of the data, which allows observing in two dimensions the cell type patterns through the samples. Finally, note that the learned representation can capture nonlinear trends in the data with a goal-oriented dense vision (cell type classification) which provides an advantage with respect to fully unsupervised models. Furthermore, the noise-filtering capabilities of the model, derived from both the topology and the sparsity-inducing priors used, can help with the visualizations [23, 51].

### Biological analysis

To further determine the potential biological relevance of the proposed DNN models, the top 10 most highly weighted nodes in the hidden layer for each output layer node were identified (i.e., the 10 top pathways for each cell type). A literature search was performed to identify published associations between the pathways and the cell types using the PubMed repository. Furthermore, the resulting networks were analyzed in the context of Gene Ontology (GO) with the Funcassociate tool [52]. Detection of statistically overrepresented GO terms was done with the hypergeometric test, using multiple-testing adjustments with the Benjamini and Hochberg false discovery rate [53] and a significance level of α = 0.05, using the FatiGO tool [54].

## Supplementary Information

**Additional file 1.** Supplementary material for: Integrating pathway knowledge with deep neural networks to reduce the dimensionality in single-cell RNA-seq data.

## Data Availability

The datasets supporting the conclusions of this article are available in the GEO repository, in the following accessions: https://www.ncbi.nlm.nih.gov/geo/query/acc.cgi?acc=GSE115978 https://www.ncbi.nlm.nih.gov/geo/query/acc.cgi?acc=GSE41265 https://www.ncbi.nlm.nih.gov/geo/query/acc.cgi?acc=GSE42268 https://www.ncbi.nlm.nih.gov/geo/query/acc.cgi?acc=GSE45719 https://www.ncbi.nlm.nih.gov/geo/query/acc.cgi?acc=GSE76483 https://www.ncbi.nlm.nih.gov/geo/query/acc.cgi?acc=GSE47835 https://www.ncbi.nlm.nih.gov/geo/query/acc.cgi?acc=GSE52583 https://www.ncbi.nlm.nih.gov/geo/query/acc.cgi?acc=GSE55291 https://www.ncbi.nlm.nih.gov/geo/query/acc.cgi?acc=GSE57249 https://www.ncbi.nlm.nih.gov/geo/query/acc.cgi?acc=GSE60297 https://www.ncbi.nlm.nih.gov/geo/query/acc.cgi?acc=GSE60361 https://www.ncbi.nlm.nih.gov/geo/query/acc.cgi?acc=GSE60768 https://www.ncbi.nlm.nih.gov/geo/query/acc.cgi?acc=GSE61470 https://www.ncbi.nlm.nih.gov/geo/query/acc.cgi?acc=GSE63576 https://www.ncbi.nlm.nih.gov/geo/query/acc.cgi?acc=GSE64960 https://www.ncbi.nlm.nih.gov/geo/query/acc.cgi?acc=GSE65525 https://www.ncbi.nlm.nih.gov/geo/query/acc.cgi?acc=GSE66202 https://www.ncbi.nlm.nih.gov/geo/query/acc.cgi?acc=GSE70844 https://www.ncbi.nlm.nih.gov/geo/query/acc.cgi?acc=GSE75107 https://www.ncbi.nlm.nih.gov/geo/query/acc.cgi?acc=GSE75108 https://www.ncbi.nlm.nih.gov/geo/query/acc.cgi?acc=GSE75109 https://www.ncbi.nlm.nih.gov/geo/query/acc.cgi?acc=GSE75110 https://www.ncbi.nlm.nih.gov/geo/query/acc.cgi?acc=GSE75111 https://www.ncbi.nlm.nih.gov/geo/query/acc.cgi?acc=GSE74923 https://www.ncbi.nlm.nih.gov/geo/query/acc.cgi?acc=GSE67120 https://www.ncbi.nlm.nih.gov/geo/query/acc.cgi?acc=GSE79510 https://www.ncbi.nlm.nih.gov/geo/query/acc.cgi?acc=GSE70605 https://www.ncbi.nlm.nih.gov/geo/query/acc.cgi?acc=GSE81682 https://www.ncbi.nlm.nih.gov/geo/query/acc.cgi?acc=GSE66578 https://www.ncbi.nlm.nih.gov/geo/query/acc.cgi?acc=GSE74596 https://www.ncbi.nlm.nih.gov/geo/query/acc.cgi?acc=GSE77029 https://www.ncbi.nlm.nih.gov/geo/query/acc.cgi?acc=GSE65924 https://www.ncbi.nlm.nih.gov/geo/query/acc.cgi?acc=GSE70657 And the ArrayExpress repository, in: https://www.ebi.ac.uk/arrayexpress/experiments/E-MTAB-2805/. Project name: Prior Knowledge-based NN. Project home page: https://github.com/babelomics/signalization_prior_knowledge_based_nn Operating system(s): tested on GNU/Linux × 68. Programming language: Python 3.6+. Other requirements: tensorflow 2+ (see environment.yml for the full specifications). License: MIT. The supporting code is available here: https://github.com/babelomics/signalization_prior_knowledge_based_nn.

## Abbreviations

AMI: adjusted mutual information
ARI: adjusted random index
CAF: cancer-associated fibroblasts
DAE: denoising autoencoder
DNA: desoxyribonucleic acid
DNN: deep neural network
GEO: gene expression omnibus
GRN: gene regulatory networks
LPGO: leave-p-groups-out
MAP: mean average precision
ML: machine learning
NN: neural network
PCA: principal component analysis
PPI: protein-protein interaction
RF: random forest
RNA-seq: RNA sequencing
scRNA-seq: single-cell RNA sequencing
SGD: stochastic gradient descent
SVM: support vector machine
TPM: transcript per million
t-SNE: t-distributed stochastic neighbor embedding
UMAP: uniform manifold approximation and projection
1-layer design: the NN with 1 hidden layer
2-layer design: the NN with 2 hidden layers

## References

[1] Olsen TK, Baryawno N (2018). Introduction to single-cell RNA sequencing. Curr Protoc Mol Biol.

[2] Wang Z, Gerstein M, Snyder M (2009). RNA-Seq: a revolutionary tool for transcriptomics. Nat Rev Genet.

[3] Quinn EM, Cormican P, Kenny EM, Hill M, Anney R, Gill M, Corvin AP, Morris DW (2013). Development of strategies for SNP detection in RNA-seq data: application to lymphoblastoid cell lines and evaluation using 1000 genomes data. PLoS One.

[4] Kunz M, Löffler-Wirth H, Dannemann M, Willscher E, Doose G, Kelso J, Kottek T, Nickel B, Hopp L, Landsberg J (2018). RNA-seq analysis identifies different transcriptomic types and developmental trajectories of primary melanomas. Oncogene.

[5] Shukla S, Evans JR, Malik R, Feng FY, Dhanasekaran SM, Cao X, Chen G, Beer DG, Jiang H, Chinnaiyan AM (2017). Development of a RNA-Seq based prognostic signature in lung adenocarcinoma. J Natl Cancer Inst.

[6] Arnold CD, Gerlach D, Stelzer C, Boryń ŁM, Rath M, Stark A (2013). Genome-wide quantitative enhancer activity maps identified by STARR-seq. Science.

[7] Conde L, Bracci PM, Richardson R, Montgomery SB, Skibola CF (2013). Integrating GWAS and expression data for functional characterization of disease-associated SNPs: an application to follicular lymphoma. Am J Hum Genet.

[8] Maher CA, Kumar-Sinha C, Cao X, Kalyana-Sundaram S, Han B, Jing X, Sam L, Barrette T, Palanisamy N, Chinnaiyan AM (2009). Transcriptome sequencing to detect gene fusions in cancer. Nature.

[9] Saliba A-E, Westermann AJ, Gorski SA, Vogel J (2014). Single-cell RNA-seq: advances and future challenges. Nucleic Acids Res.

[10] Angerer P, Simon L, Tritschler S, Wolf FA, Fischer D, Theis FJ (2017). Single cells make big data: new challenges and opportunities in transcriptomics. Curr Opin Syst Biol.

[11] Falco MM, Peña-Chilet M, Loucera C, Hidalgo MR, Dopazo J (2020). Mechanistic models of signaling pathways deconvolute the glioblastoma single-cell functional landscape. NAR Cancer.

[12] Poulin J-F, Tasic B, Hjerling-Leffler J, Trimarchi JM, Awatramani R (2016). Disentangling neural cell diversity using single-cell transcriptomics. Nat Neurosci.

[13] Darmanis S, Sloan SA, Zhang Y, Enge M, Caneda C, Shuer LM, Gephart MGH, Barres BA, Quake SR (2015). A survey of human brain transcriptome diversity at the single cell level. Proc Natl Acad Sci.

[14] Pierson E, Yau C (2015). ZIFA: dimensionality reduction for zero-inflated single-cell gene expression analysis. Genome Biol.

[15] Tsuyuzaki K, Sato H, Sato K, Nikaido I (2020). Benchmarking principal component analysis for large-scale single-cell RNA-sequencing. Genome Biol.

[16] Becht E, McInnes L, Healy J, Dutertre C-A, Kwok IW, Ng LG, Ginhoux F, Newell EW (2019). Dimensionality reduction for visualizing single-cell data using UMAP. Nat Biotechnol.

[17] Van der Maaten L, Hinton G. Visualizing data using t-SNE. J Mach Learn Res. 2008;9:–11.

[18] Kobak D, Berens P (2019). The art of using t-SNE for single-cell transcriptomics. Nat Commun.

[19] Kobak D, Linderman GC (2021). Initialization is critical for preserving global data structure in both t-SNE and UMAP. Nat Biotechnol.

[20] Hu H, Li Z, Li X, Yu M, Pan X. ScCAEs: deep clustering of single-cell RNA-seq via convolutional autoencoder embedding and soft K-means. Brief Bioinforma. 2021:bbab321. 10.1093/bib/bbab321 34472585

[21] Tian T, Wan J, Song Q, Wei Z (2019). Clustering single-cell RNA-seq data with a model-based deep learning approach. Nat Mach Intell.

[22] Ding J, Condon A, Shah SP (2018). Interpretable dimensionality reduction of single cell transcriptome data with deep generative models. Nat Commun.

[23] Lin C, Jain S, Kim H, Bar-Joseph Z (2017). Using neural networks for reducing the dimensions of single-cell RNA-Seq data. Nucleic Acids Res.

[24] Kiselev VY, Yiu A, Hemberg M (2018). scmap: projection of single-cell RNA-seq data across data sets. Nat Methods.

[25] Crawford J, Greene CS (2020). Incorporating biological structure into machine learning models in biomedicine. Curr Opin Biotechnol.

[26] Kanehisa M, Furumichi M, Tanabe M, Sato Y, Morishima K (2017). KEGG: new perspectives on genomes, pathways, diseases and drugs. Nucleic Acids Res.

[27] Hao J, Masum M, Oh JH, Kang M. Gene-and Pathway-Based Deep Neural Network for Multi-omics Data Integration to Predict Cancer Survival Outcomes. In: Cai Z., Skums P., Li M. (eds) Bioinformatics Research and Applications. ISBRA 2019. Lecture Notes in Computer Science: Springer; 2019;11490:113–24. 10.1007/978-3-030-20242-2_10.

[28] Hao J, Kim Y, Kim T-K, Kang M (2018). PASNet: pathway-associated sparse deep neural network for prognosis prediction from high-throughput data. BMC Bioinformatics.

[29] Li C, Liu B, Kang B, Liu Z, Liu Y, Chen C, Ren X, Zhang Z (2020). SciBet as a portable and fast single cell type identifier. Nat Commun.

[30] Kimmerling RJ, Szeto GL, Li JW, Genshaft AS, Kazer SW, Payer KR, de Riba BJ, Blainey PC, Irvine DJ, Shalek AK (2016). A microfluidic platform enabling single-cell RNA-seq of multigenerational lineages. Nat Commun.

[31] Jerby-Arnon L, Shah P, Cuoco MS, Rodman C, Su M-J, Melms JC, Leeson R, Kanodia A, Mei S, Lin J-R (2018). A cancer cell program promotes T cell exclusion and resistance to checkpoint blockade. Cell.

[32] Carroll MC (2004). The complement system in B cell regulation. Mol Immunol.

[33] Kirk SJ, Cliff JM, Thomas JA, Ward TH (2010). Biogenesis of secretory organelles during B cell differentiation. J Leukoc Biol.

[34] Song G, Ouyang G, Bao S (2005). The activation of Akt/PKB signaling pathway and cell survival. J Cell Mol Med.

[35] Vergadi E, Ieronymaki E, Lyroni K, Vaporidi K, Tsatsanis C (2017). Akt signaling pathway in macrophage activation and M1/M2 polarization. J Immunol.

[36] Wu Y, Humphrey MB, Nakamura MC (2008). Osteoclasts—the innate immune cells of the bone. Autoimmunity.

[37] Habibi L, Ebtekar M, Jameie S (2009). Immune and nervous systems share molecular and functional similarities: memory storage mechanism. Scand J Immunol.

[38] Dustin ML (2012). Signaling at neuro/immune synapses. J Clin Invest.

[39] Dustin ML (2014). The immunological synapse. Cancer Immunol Res.

[40] Maurer S, Wabnitz GH, Kahle NA, Stegmaier S, Prior B, Giese T, Gaida MM, Samstag Y, Hänsch GM (2015). Tasting Pseudomonas aeruginosa biofilms: human neutrophils express the bitter receptor T2R38 as sensor for the quorum sensing molecule N-(3-oxododecanoyl)-l-homoserine lactone. Front Immunol.

[41] Liu S, Xu M, Zhu C, Zhao Q, Zhou F (2018). Taste receptor T1R1/T1R3 promotes the tumoricidal activity of hepatic CD49a+ CD49b− natural killer cells. Eur J Immunol.

[42] Grassin-Delyle S, Salvator H, Mantov N, Abrial C, Brollo M, Faisy C, Naline E, Couderc L-J, Devillier P (2019). Bitter taste receptors (TAS2Rs) in human lung macrophages: receptor expression and inhibitory effects of TAS2R agonists. Front Physiol.

[43] SciBet. http://scibet.cancer-pku.cn/document.html. Accessed 15 Feb 2021.

[44] Clough E, Barrett T. The gene expression omnibus database. Methods Mol Biol. 2016;1418:93–110. 10.1007/978-1-4939-3578-9_5. 10.1007/978-1-4939-3578-9_5 PMC4944384 27008011

[45] Papatheodorou I, Moreno P, Manning J, Fuentes AM-P, George N, Fexova S, Fonseca NA, Füllgrabe A, Green M, Huang N (2020). Expression atlas update: from tissues to single cells. Nucleic Acids Res.

[46] Cao Z-J, Wei L, Lu S, Yang D-C, Gao G (2020). Searching large-scale scRNA-seq databases via unbiased cell embedding with cell BLAST. Nat Commun.

[47] Wagner GP, Kin K, Lynch VJ (2012). Measurement of mRNA abundance using RNA-seq data: RPKM measure is inconsistent among samples. Theory Biosci.

[48] Tensorflow. https://www.tensorflow.org/about/bib. Accessed 10 Jan 2021

[49] Glorot X, Bengio Y (2010). Understanding the difficulty of training deep feedforward neural networks. Proceedings of the thirteenth international conference on artificial intelligence and statistics.

[50] Li L, Jamieson K, DeSalvo G, Rostamizadeh A, Talwalkar A (2017). Hyperband: a novel bandit-based approach to hyperparameter optimization. J Mach Learn Res.

[51] Fortelny N, Bock C (2020). Knowledge-primed neural networks enable biologically interpretable deep learning on single-cell sequencing data. Genome Biol.

[52] Berriz GF, King OD, Bryant B, Sander C, Roth FP (2003). Characterizing gene sets with FuncAssociate. Bioinformatics.

[53] Benjamini Y, Hochberg Y (1995). Controlling the false discovery rate: a practical and powerful approach to multiple testing. J R Stat Soc Ser B.

[54] Al-Shahrour F, Diaz-Uriarte R, Dopazo J (2004). FatiGO: a web tool for finding significant associations of gene ontology terms with groups of genes. Bioinformatics.

