# Supplementary material for: Integrating pathway knowledge with deep neural networks to reduce the dimensionality in single-cell RNA-seq data
Source: BioData Min. 2022 Jan 3;15:1. doi: 10.1186/s13040-021-00285-4 (PMC8722116; doi:10.1186/s13040-021-00285-4)
Supplement: Supplementary file 1 — Additional file 1. Supplementary material for: Integrating pathway knowledge with deep neural networks to reduce the dimensionality in single-cell RNA-seq data. [file 13040_2021_285_MOESM1_ESM.pdf]

Supplementary material for:

Integrating pathway knowledge with deep neural networks to reduce the dimensionality in single-cell RNA-seq data

Peling Gundogdu, Carlos Loucera, Inmaculada Alamo, Joaquin Dopazo and Isabel Nepomuceno

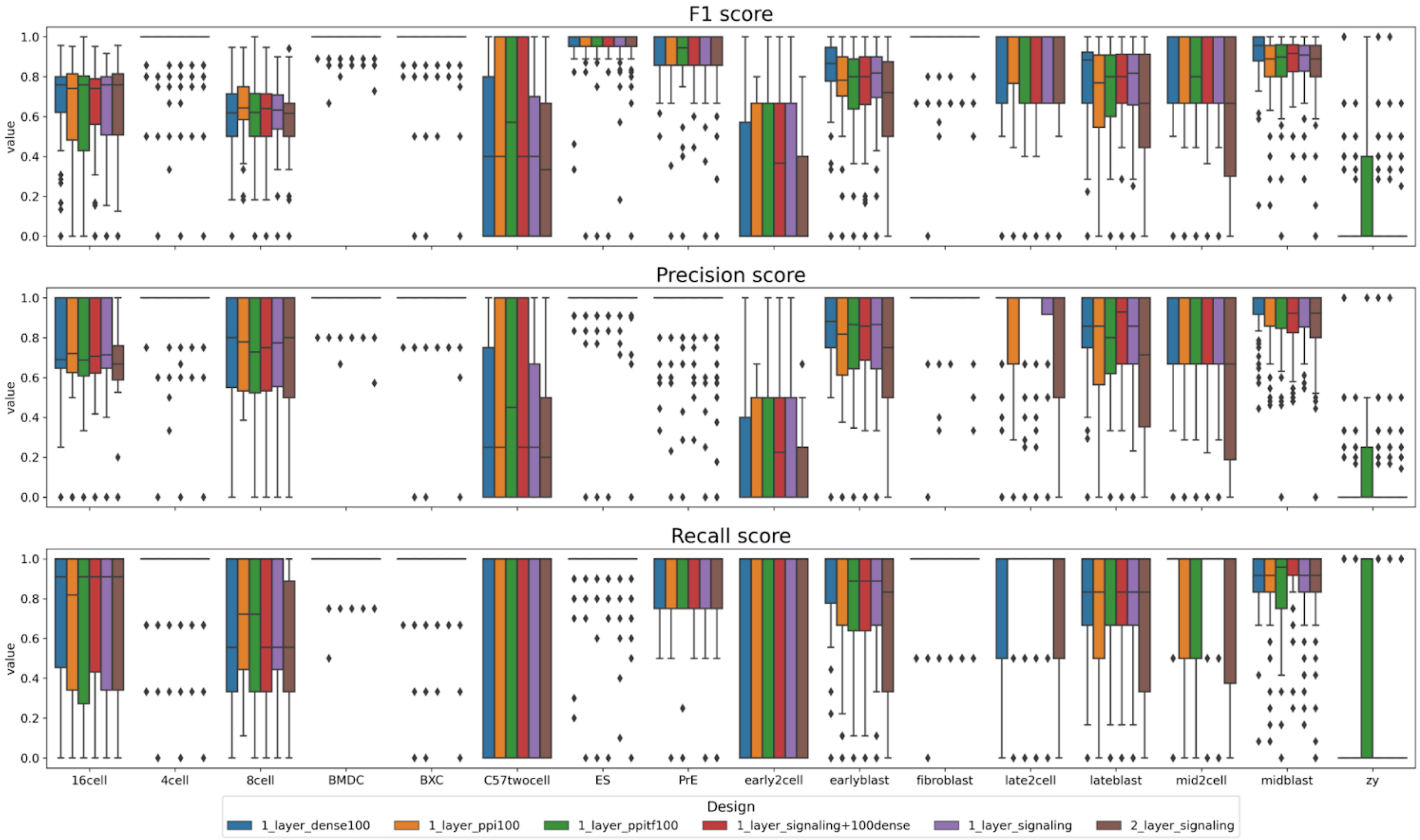

Supplementary Figure 1. Per-class metric distributions

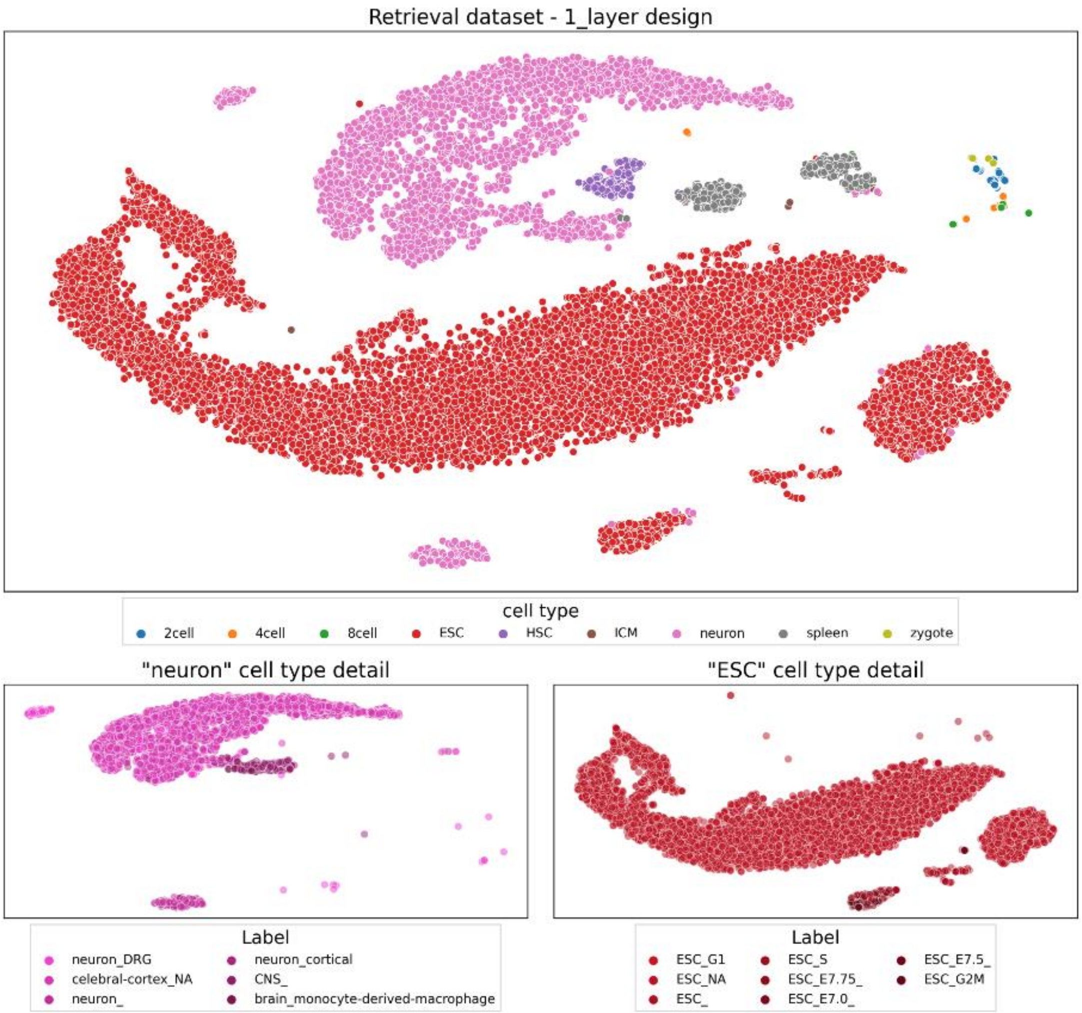

Supplementary Figure 2. Encoding information of the network with mouse cells

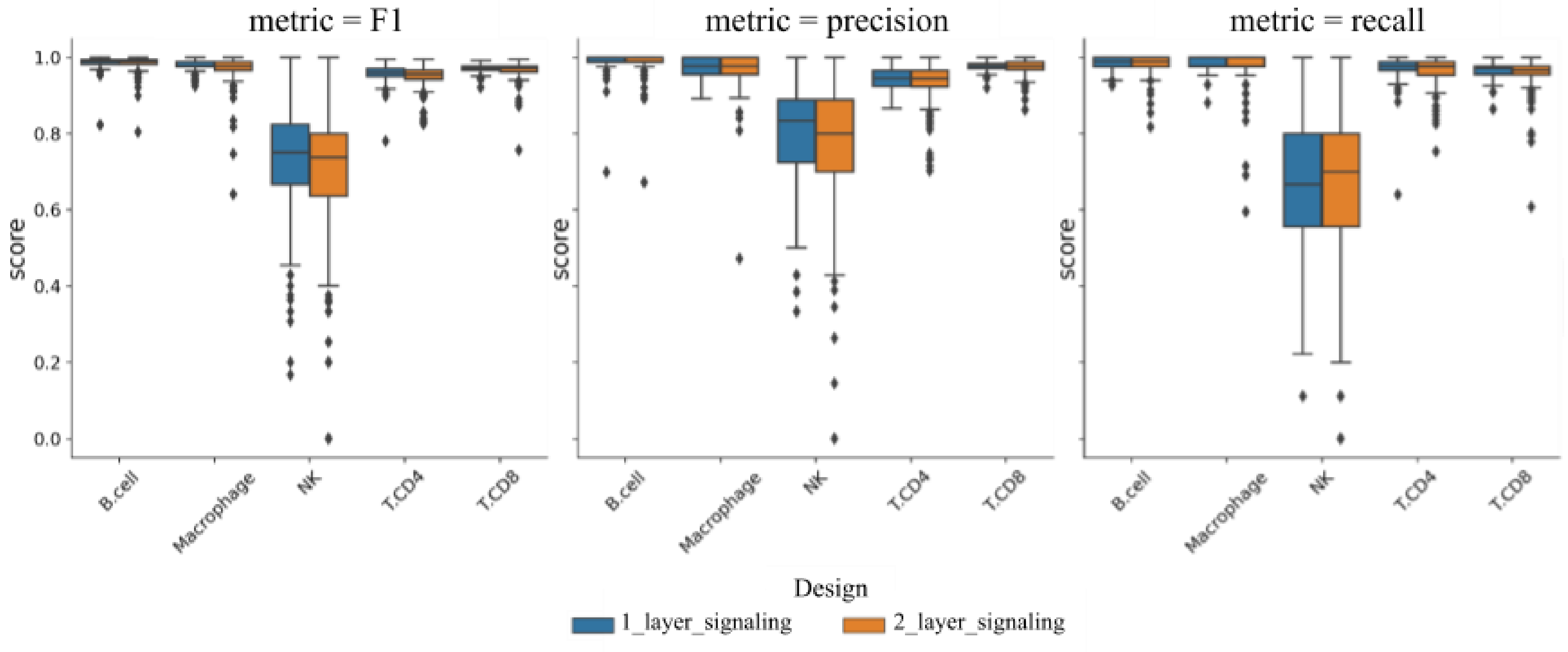

Supplementary Figure 3. The proposed network performance for per-class

Supplementary Table 1. Accession number of each experiment

Num	doi	Accession number	Experiment	Dataset	Cell type
1	10.1016/j.cell.2018.09.006	GSE115978	Melanoma	Training & testing	B.cell, T.CD4, T.CD8, Macrophage, NK, CAF, Endothelial cells, Maligant, T.cell
2	10.1038/nature12172	GSE41265	Mouse	learning	BMDC
3	10.1186/gb-2013-14-4-r31	GSE42268			ESC
4	10.1126/science.1245316	GSE45719			Embryonic cells
6	10.1038/nbt.3102	E-MTAB-2805	Mouse	retrieval	ESC
7	10.1186/s13059-016-0950-z	GSE76483			DRG
8	10.1073/pnas.1402030111	GSE47835			ESC MEF
9	10.1038/nature13173	GSE52583			distal lung epithelium
10	10.1016/j.stem.2014.11.005	GSE55291			iPS TTF ESC
11	10.1101/gr.177725.114	GSE57249			embryonic cells
12	10.1101/gr.171645.113	GSE60297			thymus TEC
13	10.1126/science.aaa1934	GSE60361			celebral-cortex
14	10.15252/msb.20156198	GSE60768			ESC NSC
15	10.1038/nbt.3154	GSE61470			ESC PS NP HF
16	10.1038/cr.2015.149	GSE63576			DRG
17	10.1016/j.cub.2015.01.034	GSE64960			granulosa
18	10.1016/j.cell.2015.04.044.	GSE65525			ESC
19	10.1016/j.devcel.2015.09.009.	GSE66202			kidney
20	10.1038/nbt.3443	GSE70844			neuron
21	10.1016/j.cell.2015.11.009	GSE75107			CNS/Th17
22	10.1016/j.cell.2015.11.009	GSE75108			LN/Th17
23	10.1016/j.cell.2015.11.009	GSE75109			spleen LN/Th17
24	10.1016/j.cell.2015.11.009	GSE75110			spleen LN/Th17
25	10.1016/j.cell.2015.11.009	GSE75111			spleen LN/Th17
26	10.1038/ncomms10220	GSE74923			cancer
27	10.1038/nature17997	GSE67120			HSC
28	10.1186/s12974-016-0581-z	GSE79510			brain
29	10.1038/celldisc.2016.10	GSE70605			embryonic cells

30	10.1182/blood-2016-05-716480	GSE81682			HSC
31	10.1172/JCI77378	GSE66578			lung
32	10.1038/ni.3437	GSE74596			thymus
33	10.1038/ni.3412	GSE77029			bone marrow
34	10.1016/j.devcel.2016.02.020	GSE65924			embryonic cells
35	10.1038/ncomms11075	GSE70657			HSC

Supplementary Table 2. Parameter values

Parameter name	Experiment	Parameter value
epochs	Human & Mouse	100
batch_size	Human & Mouse	10
kernel_initializer	Human & Mouse	glorot_uniform
bias_initializer	Human & Mouse	zeros
activation	Mouse	tanh (hidden layer) / softmax (last layer)
optimizer	Mouse	SGD
activation	Human	relu (hidden layer) / softmax (last layer)
optimizer	Human	Adam

Supplementary Table 3. Cell type and number of samples detail of mouse datasets

cell type	Number of samples *learning* dataset	Number of samples *retrieval* dataset
16cell	54	-
4cell	14	55
8cell	47	53
BMDC	18	-
BXC	13	-
C57twocell	8	-
ES	49	-
PrE	22	-
early2cell	8	-
earlyblast	43	-
fibroblast	10	-
late2cell	10	-
lateblast	30	-
mid2cell	12	-
midblast	60	-
zy	4	-
2cell	-	85
ESC	-	9,123
HSC	-	202
ICM	-	22
neuron	-	3,667
spleen	-	420
zygote	-	18

Supplementary Table 4. Clustering performance, Homo: Homogeneity, Comp: Completeness, V-m: V-measure, ARI: Adjusted Rand Index, AMI: Adjusted Mutual Information, FM: Fowlkes-Mallows, Avg.: the mean of all metrics

P-Groups-Out	Architecture	# of nodes (Layer2)	Homo	Comp	V-m	ARI	AMI	FM	Avg.
2 cell types	Dense	X	0.909	0.895	0.900	0.906	0.897	0.961	0.911
	Dense with signaling pathways	X	0.959	0.955	0.957	0.965	0.955	0.983	0.962
	Dense with PPI	X	0.925	0.912	0.917	0.922	0.915	0.969	0.927
	Dense with PPI/TF	X	0.925	0.912	0.917	0.922	0.915	0.969	0.927
	Signaling pathways	X	0.945	0.936	0.940	0.953	0.939	0.979	0.949
		100	0.946	0.939	0.943	0.953	0.941	0.978	0.950
4 cell types	Dense	X	0.801	0.799	0.798	0.725	0.786	0.814	0.787
	Dense with signaling pathways	X	0.804	0.797	0.798	0.718	0.786	0.811	0.786
	Dense with PPI	X	0.811	0.804	0.805	0.728	0.794	0.817	0.793
	Dense with PPI/TF	X	0.820	0.808	0.812	0.746	0.802	0.827	0.802
	Signaling pathways	X	0.797	0.788	0.790	0.716	0.778	0.809	0.780
		100	0.775	0.803	0.786	0.729	0.774	0.820	0.781
6 cell types	Dense	X	0.796	0.743	0.768	0.612	0.753	0.707	0.730
	Dense with signaling pathways	X	0.784	0.733	0.756	0.604	0.741	0.701	0.720
	Dense with PPI	X	0.796	0.747	0.769	0.631	0.755	0.720	0.737
	Dense with PPI/TF	X	0.800	0.743	0.769	0.613	0.755	0.708	0.732
	Signaling pathways	X	0.770	0.723	0.744	0.603	0.729	0.699	0.711
		100	0.750	0.699	0.723	0.573	0.705	0.677	0.688
8 cell types	Dense	X	0.806	0.757	0.780	0.605	0.763	0.677	0.731
	Dense with signaling pathways	X	0.798	0.761	0.778	0.610	0.761	0.683	0.732
	Dense with PPI	X	0.793	0.759	0.775	0.596	0.757	0.673	0.725
	Dense with PPI/TF	X	0.804	0.768	0.784	0.611	0.768	0.686	0.737
	Signaling pathways	X	0.766	0.742	0.752	0.572	0.733	0.653	0.703
		100	0.744	0.718	0.729	0.549	0.708	0.635	0.681

Supplementary Table 5. Cell type and number of samples detail of melanoma datasets

	Number of samples	
cell type	*training* dataset	*testing* dataset
B.cell	573	245
Macrophage	294	126
NK	64	28
Neg.cell	-	2,228
T.CD4	599	257
T.CD8	1,231	528

Supplementary Table 6. Top 10 most highly weighted pathways for NK

Cell type	Pathway ID	Pathway name
NK	hsa04664	Fc epsilon RI signaling pathway
	hsa04742	Taste transduction
	hsa05100	Bacterial invasion of epithelial cells
	hsa04726	Serotonergic synapse
	hsa04022	cGMP-PKG signaling pathway
	hsa03320	PPAR signaling pathway
	hsa04066	HIF-1 signaling pathway
	hsa04914	Progesterone-mediated oocyte maturation
	hsa04150	mTOR signaling pathway
	hsa04713	Circadian entrainment
TCD8	hsa05100	Bacterial invasion of epithelial cells
	hsa04664	Fc epsilon RI signaling pathway
	hsa04742	Taste transduction
	hsa04022	cGMP-PKG signaling pathway
	hsa04150	mTOR signaling pathway
	hsa04915	Estrogen signaling pathway
	hsa04713	Circadian entrainment
	hsa04914	Progesterone-mediated oocyte maturation
	hsa04012	ErbB signaling pathway
	hsa04670	Leukocyte transendothelial migration
TCD4	hsa04664	Fc epsilon RI signaling pathway
	hsa05100	Bacterial invasion of epithelial cells
	hsa04742	Taste transduction
	hsa04726	Serotonergic synapse
	hsa04973	Carbohydrate digestion and absorption
	hsa04650	Natural killer cell mediated cytotoxicity
	hsa04919	Thyroid hormone signaling pathway
	hsa04012	ErbB signaling pathway
	hsa04670	Leukocyte transendothelial migration
	hsa04666	Fc gamma R-mediated phagocytosis
BCell	hsa04150	mTOR signaling pathway
	hsa04022	cGMP-PKG signaling pathway

	hsa04726	Serotonergic synapse
	hsa04915	Estrogen signaling pathway
	hsa04650	Natural killer cell mediated cytotoxicity
	hsa04960	Aldosterone-regulated sodium reabsorption
	hsa04972	Pancreatic secretion
	hsa04914	Progesterone-mediated oocyte maturation
	hsa04610	Complement and coagulation cascades
	hsa04913	Ovarian steroidogenesis
Macrophage	hsa05100	Bacterial invasion of epithelial cells
	hsa04664	Fc epsilon RI signaling pathway
	hsa04742	Taste transduction
	hsa04022	cGMP-PKG signaling pathway
	hsa04726	Serotonergic synapse
	hsa04915	Estrogen signaling pathway
	hsa04151	PI3K-Akt signaling pathway
	hsa04380	Osteoclast differentiation
	hsa04973	Carbohydrate digestion and absorption
	hsa04650	Natural killer cell mediated cytotoxicity
